# A commentary on “Magnetic sphincter augmentation in the management of gastro-esophageal reflux disease: a systematic review and meta-analysis”

**DOI:** 10.1097/JS9.0000000000002990

**Published:** 2025-07-08

**Authors:** Huayang Pang, Jinlai Wei, Xiufeng Chen

**Affiliations:** a Department of Gastrointestinal Cancer Center, Chongqing University Cancer Hospital, Chongqing, China; b Department of Gastrointestinal Surgery, the First Affiliated Hospital of Chongqing Medical University, Chongqing, China

*Dear Editor,*

We read with great interest the study conducted by Fadel et al^[1]^, which investigated the efficacy, quality of life, and safety of magnetic sphincter augmentation (MSA) compared to fundoplication in managing patients with gastro-esophageal reflux disease (GERD). In this comprehensive meta-analysis comprising 39 studies involving 8075 patients, the authors demonstrated that MSA is a safe and effective procedure for reducing symptom burden of GERD and can potentially improve patient satisfaction and functional outcomes. The authors should be commended for providing evidence supporting the superiority of MSA in GERD management. However, there are several aspects that necessitate further clarification before implementing these findings into clinical practice.

Firstly, a comprehensive literature search is a crucial prerequisite for ensuring the reliability of a meta-analysis. In this study, despite conducting searches across multiple representative databases with the assistance of a librarian, we identified three important studies (Khaitan et al^[2]^, Patel et al^[3]^, and Wu et al^[4]^) that were omitted by the authors. Upon careful evaluation of their full texts, we determined that these studies indeed met the inclusion criteria for this meta-analysis; therefore, it would have been appropriate for the authors to include them in their analysis.

Secondly, in describing the characteristics of included studies, the authors demonstrated that there were 12 prospective studies included in the meta-analysis. However, only one reference was cited by the authors.

Lastly, in this study, a significant level of heterogeneity was observed in the results of the majority of meta-analyses, which poses substantial implications for result reliability. However, it is regrettable that the authors did not conduct further analysis and elucidation on these variations. Therefore, we strongly recommend that the authors undertake subgroup analysis, meta-regression, and other methods to explore the origins of heterogeneity and identify potential GERD patients who are truly suitable for MSA.

Overall, we thank Fadel and colleagues from the bottom of our hearts for their concerted efforts in investigating the clinical value of MSA in patients with GERD. They have made an important step forward on this clinically meaningful topic. But we just point out our concerns, which could potentially assist the authors in enhancing the clarity of their findings.

This study is compliant with the TITAN Guidelines 2025^[5]^.

## Data Availability

No data used in this letter to the editor.

## Research registration unique identifying number (UIN)

Not required for this letter to the editor.
